# Transcriptome- and methylome-wide association studies prioritized lung cancer genes in East Asian populations

**DOI:** 10.1371/journal.pgen.1012332

**Published:** 2026-11-06

**Authors:** Xingjie Hao, Minghan Qu, Yi Jiang, Cheng Wang, Ziwei Zhu, Pinpin Long, Chaolong Wang, Hongxia Ma, Shanshan Cheng

**Affiliations:** 1 Department of Epidemiology and Biostatistics, School of Public Health, Tongji Medical College, Huazhong University of Science and Technology, Wuhan, Hubei, China; 2 Department of Neonatology, Fujian Children’s Hospital (Fujian Branch of Shanghai Children’s Medical Center), College of Clinical Medicine for Obstetrics & Gynecology and Pediatrics, Fujian Medical University, Fuzhou, Fujian, China; 3 Department of Epidemiology, Center for Global Health, School of Public Health, Nanjing Medical University, Nanjing, Jiangsu, China; 4 Department of Occupational and Environmental Health, School of Public Health, Tongji Medical College, Huazhong University of Science and Technology, Wuhan, Hubei, China; Newcastle University, UNITED KINGDOM OF GREAT BRITAIN AND NORTHERN IRELAND

## Abstract

The relationship between gene expression, DNA methylation, and lung cancer remains elusive in East Asian (EAS) populations. Transcriptome- and methylome-wide association studies (TWAS/MWAS) help uncover gene–trait associations by integrating multi-omics data, yet models trained mainly on European references limit cross-population generalizability. To address this, we developed EAS-specific gene expression and DNA methylation prediction models using whole-blood data from over 800 and 4,300 individuals, respectively. Applying these to lung cancer GWAS from Nanjing Medical University (13,327 cases; 13,328 controls) and Biobank Japan (4,444 cases; 489,012 controls), we identified 25 genes and 42 CpGs associated with lung cancer risk in the discovery stage, with seven genes and 32 CpGs replicated. Three novel genes outside known GWAS loci—*RP11-57K17.1* (6q22.31), *RP11-313J2.1* (10q11.21), and *BMS1P2* (10q11.22)—were identified. In addition, Mendelian randomization analyses suggested that methylation-mediated downregulation of *RP11-147L13.11* can reduce lung cancer risk. Our study reveals new susceptibility genes and offers insights into genetic-epigenetic regulatory mechanisms in lung cancer among EAS populations.

## Author summary

This study leveraged large-scale blood multi-omics datasets to conduct comprehensive data mining and analysis for lung cancer in East Asian (EAS) populations. Using two-stage transcriptome- and methylome-wide association analyses (TWAS/MWAS), this study screened and validated 7 genes and 32 CpG sites associated with lung cancer risk, including three newly found genes. Subsequent colocalization and Mendelian randomization analyses revealed key epigenetic regulatory pathways. Specifically, two methylation markers were found to regulate the lncRNA *RP11-147L13.11* and ultimately affect lung cancer susceptibility. This data-driven work uncovers population-specific molecular markers and regulatory mechanisms, offering solid clues for further research on the onset and progression of lung cancer among East Asians.

## Introduction

Lung cancer is the most frequently diagnosed cancer and the leading cause of cancer-related death both in China and globally [1,2]. In addition to the well-established environmental risk factors such as tobacco smoking, genetic factors also play a critical role in the etiology of lung cancer [3]. Our current understanding of the genetic architecture of lung cancer has primarily been shaped by genome-wide association studies (GWAS), which have collectively identified 51 susceptibility loci to date [4–8]. However, the majority of these loci are located in the non-coding regions, thus posing significant challenges in elucidating their functional consequences and underlying biological mechanisms.

Recently, transcriptome-wide association study (TWAS) has been broadly used in the field of complex traits study to prioritize susceptibility genes due to its high statistical power and interpretability [9–11]. Bosse et al. and Zhu et al. utilized TWAS to uncover several novel genes for lung cancer, thereby complementing current GWAS findings [12,13]. Nevertheless, the prediction models employed in the TWAS framework were all derived from expression reference panels of European (EUR) populations. When applied to other populations, the prediction accuracy and statistical power of these EUR-trained models may diminish due to the differences in genetic architecture, allele frequency, and linkage disequilibrium (LD) across populations [10,14–16]. Therefore, it is imperative to conduct TWAS using ancestry-matched prediction models [17,18]. However, the scarcity of large-scale non-European expression datasets with individual-level data has hindered the development of ancestry-specific prediction models for other populations. Recently, Zhang et al. proposed a method that enables the construction of expression prediction models using only summary-level expression panels [19], which are often publicly available with larger sample sizes. This innovative approach offers a promising alternative for building effective ancestry-specific models.

Despite the success of TWAS in identifying the candidate genes, only a small fraction of GWAS loci have been mapped to gene level, suggesting that additional mechanisms beyond gene expression contribute to the genetic susceptibility of lung cancer [12,13]. DNA methylation (DNAm), a key epigenetic modification, can regulate gene expression and may play a critical role in cancer development. Epigenome-wide association study (EWAS) showed that many DNAm sites are associated with lung cancer risk [20–23]. However, EWAS often suffer from limitations such as insufficient statistical power due to small sample sizes, selection bias, and reverse causality, resulting in inconsistent findings across studies [21–23]. One potential solution to address such limitations is to perform a methylome-wide association study (MWAS), which links traits to genetically predicted methylation levels at CpG sites [24–26]. Similar to TWAS, the current prediction models in MWAS framework have predominantly been constructed based on EUR populations, which may introduce potential bias when directly applied to East Asian (EAS) GWAS datasets.

In the current study, we conducted EAS-specific TWAS and MWAS for lung cancer by integrating two lung cancer cohorts with multi-omics reference dataset in EAS populations. Our final goal is to achieve a comprehensive understanding of genetic susceptibility to lung cancer with interpretable functional insights in the EAS population.

## Methods

### Ethics statement

This study was approved by the Ethics and Human Subject Committee of Tongji Medical College, Huazhong University of Science and Technology. All participants provided written informed consent.

### Study design

The overall study design is illustrated in Fig 1. We first constructed EAS-specific expression and methylation prediction models. The expression prediction models were based on a meta-analysis of three Japanese expression QTL (eQTL) summary data [27–29] and the methylation prediction models were based on mQTL data of the Dongfeng-Tongji (DFTJ) cohort [30]. Then we performed a two-stage TWAS and MWAS of lung cancer in EAS populations, including the discovery stage using Nanjing Medical University (NJMU) lung cancer GWAS dataset [5] and the validation stage using Biobank Japan (BBJ) lung cancer GWAS dataset [31]. For the replicated genes and CpGs, we further applied a three-step summary-based Mendelian randomization (SMR) analysis to explore the regulatory pathways they are involved in, as well as their potential contributions to lung cancer development.

**Fig 1:**
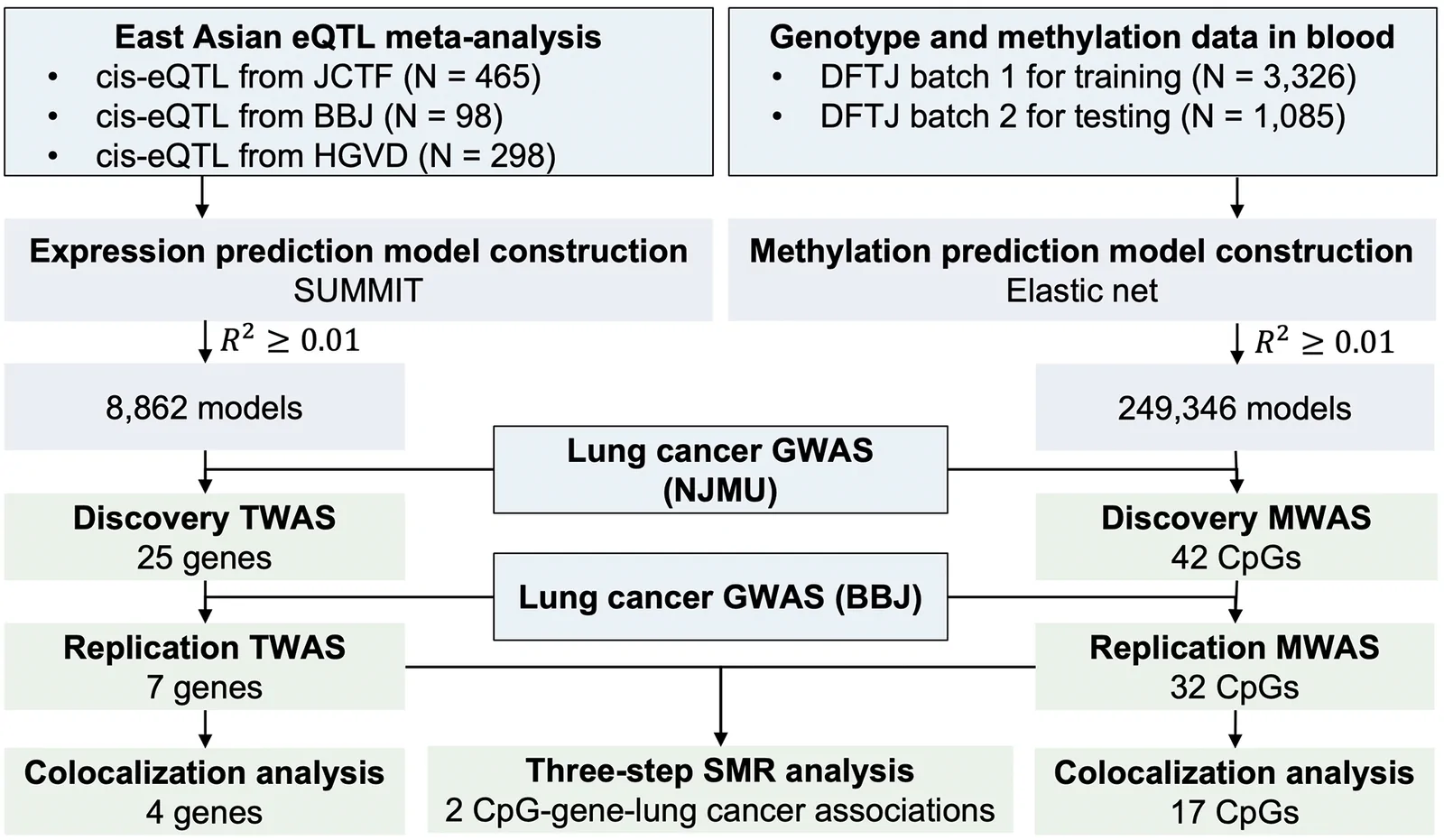
Overall workflow of the study. Abbreviations: BBJ, Biobank Japan; CpG, cytosine-phosphoguanine; DFTJ, Dongfeng-Tongji; DNAm, DNA methylation; eQTL, expression quantitative trait loci; GWAS, Genome-wide association studies; HGVD, Human Genetic Variation Database; JCTF, Japan COVID-19 Task Force; MWAS, Methylome-wide association studies; NJMU, Nanjing Medical University; SMR, summary data-based Mendelian randomization; SUMMIT, the Summary-level Unified Method for Modeling Integrated Transcriptome; LASSO, Least Absolute Shrinkage and Selection Operator; ElNet, Elastic Net; MCP, Minimax Concave Penalty; SCAD, Smoothly Clipped Absolute Deviation; MNet, Elastic Net with MCP penalty; TWAS, Transcriptome-wide association studies.

### Construction of gene expression prediction model using EAS eQTL summary data

To enhance the predictive power of our models, we aggregated publicly available EAS eQTL summary datasets, including three Japanese studies: the Human Genetic Variation Database (HGVD, N = 298) [27], the BBJ (N = 98) [28], and the Japan COVID-19 Task Force (JCTF, N = 465) [29]. Gene expression levels were measured in whole blood by Agilent microarray in HGVD (with association between ~ 1.3 million SNPs and 19,477 genes), by RNA-Seq in JCTF (with association between ~ 4.0 million SNPs and 19,251 genes) and in BBJ (with association between ~ 4.8 million SNPs and 23,015 genes). We performed an inverse variance weighted fixed-effect meta-analysis of these three eQTL datasets by METAL software [32], yielding associations between 29,080 genes and ~ 5.5 million SNPs with minor allele frequency (MAF) > 0.01.

We applied the Summary-level Unified Method for Modeling Integrated Transcriptome (SUMMIT), an advanced method that utilizes summary-level eQTL data, to construct a prediction model for each gene [19]. Specifically, SUMMIT employs a linear regression model to represent the relationship between gene expression levels and nearby cis-SNPs. Various penalty terms, such as Least Absolute Shrinkage and Selection Operator (LASSO), Elastic Net (ElNet), Minimax Concave Penalty (MCP), Smoothly Clipped Absolute Deviation (SCAD), and Elastic Net with MCP penalty (MNet), were incorporated into the model for penalized regression to constrain the regression coefficients. The objective function was simplified to include the marginal effect size of cis-eQTL, LD matrix, and primary penalty term, requiring only eQTL summary data. The LD matrix was estimated using EAS samples from 1000 Genomes Project (1KGP) [33] via a shrinkage estimator to enhance stability by reducing noise. To improve the accuracy of the genetic models, we further imputed the SNPs in the lung cancer GWAS summary statistics (see the section “Lung cancer TWAS in EAS populations” for details) to be included in model construction, thereby expanding the overlap with the genetic variants included in the models [34]. This was done via the Gaussian summary statistics imputation (ImpG) method [35] implemented by the FIZI software (https://github.com/bogdanlab/fizi) to impute missing SNP z-scores using EAS samples from 1KGP [33] as reference.

### Evaluating gene expression prediction models using Shiyan multi-omics data

We utilized both genotype and gene expression data from the Shiyan multi-omics dataset for parameter tuning and model validation [36,37]. Participants from the Shiyan cohort were recruited in 2021 from the general population in Shiyan, Hubei Province. All participants underwent routine physical examinations and provided blood and urine samples for multi-omics profiling, including genotype, DNA methylation, transcriptomics, and proteomics. In this study, a total of 160 individuals with balanced distributions of age and sex, and complete sequencing data were selected as the study sample. Written informed consent was obtained from all participants.

Genotype data were assayed using the Infinium OmniZhongHua-8 v1.4 arrays (Illumina, USA). SNPs were removed if they had a call rate < 0.95, minor allele count < 3, minor allele frequency (MAF) < 0.01, or Hardy Weinberg equilibrium *P* < 10^−6^. Samples were excluded if they had a discordance rate at duplicate sites > 0.05, call rate < 0.9, inbreeding coefficient <−0.1 or > 0.3 based on autosomal SNPs or <−0.2 based on the X chromosome, or if they were duplicated samples, or sex-mismatched samples. Following initial quality control, imputation was performed using Minimac4 [38] with reference EAS samples from 1KGP [33] and the SG10K project [39]. SNPs with an imputation quality score (*R*²) < 0.3 or a MAF < 0.01 were further excluded. Finally, a total of 159 samples and approximately 7.6 million SNPs were included in the downstream analyses.

Gene expression profiles in leukocytes were sequenced using NovaSeq6000 (Illumina, USA). Details of the laboratory methods have been previously described [37]. Raw sequencing data were processed for adapter clipping, alignment, and quantification following standard protocols with Cutadapt [40], STAR [41], and HTseq-count [42]. The expression of genes with fragments per kilobase million (FPKM) > 0 in at least 50% of the samples were kept and were further adjusted for sex, three genotype principal components (PCs), and 30 PEER factors [43]. The residuals were used as processed expression levels for subsequent analysis. After excluding one sample lacking GWAS data, 159 samples were retained for model evaluation.

We performed optimization using a coordinate descent algorithm, iteratively updating each coefficient to maximize the prediction *R*^*2*^ values (the squared correlation between the predicted and measured expressions) to select the best tuning parameters [19]. For each gene, five prediction models were constructed using five penalized regression methods. Genes for which at least one of these models had *R*^2^ ≥ 0.01 were included for further association analysis.

### Comparison of gene expression prediction models across ancestries

To evaluate the cross-ancestry transferability of gene expression prediction models, we compared EAS-specific models with EUR-specific models using the same Shiyan multi-omics dataset. For EUR-specific models, we used gene expression prediction models developed using the SUMMIT method based on cis-eQTL summary data from the eQTLGen consortium (N = 31,684) [19]. The detailed information for the models has been described elsewhere [19]. A total of 16,884 gene expression models were downloaded from the public repository (https://doi.org/10.17605/OSF.IO/7MXSA) for downstream analysis. Both the EUR and EAS models were applied to the same Shiyan dataset to impute gene expression levels. The prediction performance was evaluated by calculating the coefficient of determination (*R*^2^). We compared model performance across ancestries in terms of the number of qualified models (*R*² ≥ 0.01) and overall *R*² distribution.

### Construction and evaluation of DNA methylation prediction models using DFTJ data

The DFTJ cohort is a population-based prospective cohort comprising retired workers from the Dongfeng Motor Corporation in Shiyan, Hubei, China [30]. The cohort was initiated between September 2008 and June 2010, during which 27,009 retired employees were initially enrolled, and later expanded in October 2013 to include a total of 38,295 participants. All participants provided written informed consent.

We obtained the individual-level genotype and DNAm data of 4,411 unrelated participants in the DFTJ cohort [37]. DNA genotyping was performed using the Infinium OmniZhongHua-8 v1.4 arrays (Illumina, USA). The quality control and imputation procedures were conducted in the same way as described above for the genotype data of the Shiyan multi-omics dataset.

DNAm in leukocytes was measured across two batches using the Infinium HumanMethylationEPIC v1.0 BeadChips (Illumina, USA). Further details about data processing can be found in the original study [37]. For construction of the methylation prediction model, genetic and DNAm data from 3,326 samples in the first batch were used for model training, and data from 1,085 samples in the second batch were used for model validation.

For each CpG site, we used the elastic net method to build the genetic prediction model for DNA methylation using the R package “glmnet” with mixing parameter α = 0.5 [9^,^ 44]. Genetic variants within a 2Mb cis-region surrounding each CpG site were incorporated into the model construction. Internal tenfold cross-validation was performed, and the *R*^2^ was calculated for an initial assessment of the predictive performance of the model. The established models were subsequently applied to the second batch of the DFTJ dataset to further evaluate the predictive performance by comparing the predicted methylation levels with the measured levels. Finally, prediction models achieving an *R*^2^ ≥ 0.01 in both the training and validation datasets were selected for subsequent association analysis, in alignment with the standard criterion used in TWAS [9] and MWAS [24].

### Lung cancer TWAS in EAS populations

To investigate associations between genetically predicted gene expression and lung cancer risk, we conducted a two-stage TWAS analysis in the EAS population. Lung cancer GWAS summary data were obtained from the NJMU study as discovery dataset [5] and from the BBJ study as replication dataset [31]. The NJMU lung cancer GWAS integrated three datasets, encompassing 13,327 lung cancer cases and 13,328 controls in total from (1) the NJMU Global Screening Array (GSA) project; (2) the NJMU GWAS project; and (3) the NJMU OncoArray project [5]. The BBJ lung cancer GWAS included 4,444 cases and 489,012 controls [31]. The association between genetically predicted gene expression and lung cancer risk was estimated using a burden-type test implemented by SUMMIT. For each gene, the *P*-values of five penalized regression models were aggregated using the Cauchy combination test to derive the final *P*-value, whereas the reported Z-scores correspond to the best-performing individual prediction model. A false discovery rate (FDR)-corrected threshold of 0.05 was used to determine statistically significant associations. Association results were combined using the inverse variance weighted fixed-effect meta-analysis in METAL [32].

### Lung cancer MWAS in EAS populations

Following the TWAS analysis framework, we conducted a two-stage MWAS using the same imputed GWAS datasets. We assessed the association between genetically predicted methylation levels and lung cancer risk using SPrediXcan [45], with a FDR-corrected significance threshold of 0.05. As with TWAS, association results were combined using meta-analysis.

### Identification of novel lung cancer risk loci

Novel lung cancer risk loci in TWAS and MWAS were defined as those located over 500 kb away from any previously known risk loci. Known risk loci included the reported lung cancer association listed in the NHGRI-EBI GWAS Catalog of human GWASs [46] (with *P* < 5 × 10^−8^), in TWAS Atlas [47], and in EWAS Atlas [48] by January 5, 2025.

### Colocalization analyses for the lung cancer risk genes or CpGs

Colocalization analyses were conducted to evaluate whether the lung cancer GWAS signal and the gene/CpG QTL within the same genomic region were likely driven by a shared causal variant, using the R package Coloc [49]. For eQTL data, the Shiyan eQTL data were meta-analyzed with the HGVD, BBJ, and JCTF eQTL datasets. For mQTL data, we used our in-house cis-mQTL results from the DFTJ cohort (4,411 individuals across two methylation batches). The details of the analysis have been described previously [37]. Colocalization analysis estimates the posterior probabilities for five different hypotheses regarding the association patterns between two traits. Among these, PP.H4 represents the posterior probability that the lung cancer GWAS signal and the gene/CpG QTL signal are consistent with sharing a common causal variant, with values ranging from 0 to 1. Higher PP.H4 values indicate stronger evidence of colocalization. In this study, PP.H4 > 0.75 was considered strong evidence of colocalization.

### Three-step summary data-based MR analyses

To explore the potential molecular mechanisms, we conducted three-step summary data-based MR analyses [50] for the TWAS-identified genes and MWAS-identified CpGs. This analysis was designed to test the hypothesis that genetic variants affect DNA methylation levels, which in turn regulate gene expression, ultimately impacting lung cancer risk. By using the lead molecular QTL as the instrument variable, this summary data-based MR approach can explore potential causal associations between molecular traits and diseases. In addition, the heterogeneity in dependent instruments (HEIDI) test was performed to distinguish linkage from pleiotropy. We used the same integrated GWAS, eQTL, and mQTL summary data for the MR analyses as in the colocalization analysis to identify (1) the association between genes and lung cancer risk; (2) the association between CpGs and lung cancer risk; (3) the association between CpGs and genes. Associations with Bonferroni-corrected *P*_SMR_ < 0.05 and *P*_HEIDI_ > 0.01 were considered to show evidence consistent with pleiotropic or potentially causal effects without heterogeneity. Associations passing all three steps provided supporting evidence that the identified DNAm site may influence lung cancer risk through genetically regulated gene expression.

## Results

### East Asian-specific gene expression and methylation prediction models

The analytical framework of this study is presented in Fig 1. Based on the results of the meta-analysis of three Japanese eQTL studies totalling 861 blood samples, we trained gene expression prediction models with ElNet, LASSO, MCP, Mnet, and SCAD implemented in SUMMIT [19]. We observed that MCP yielded the largest number of qualified expression prediction models (*R*^2^ ≥ 0.01, *N* = 8,667) among the five training methods (Fig 2A and S1 Table). Among the qualified prediction models obtained by each method, those trained using ElNet exhibited the highest median *R*^2^ of 0.028. After retaining genes with at least one qualified expression prediction model and excluding those in the MHC region, 8,862 genes remained for TWAS.

**Fig 2:**
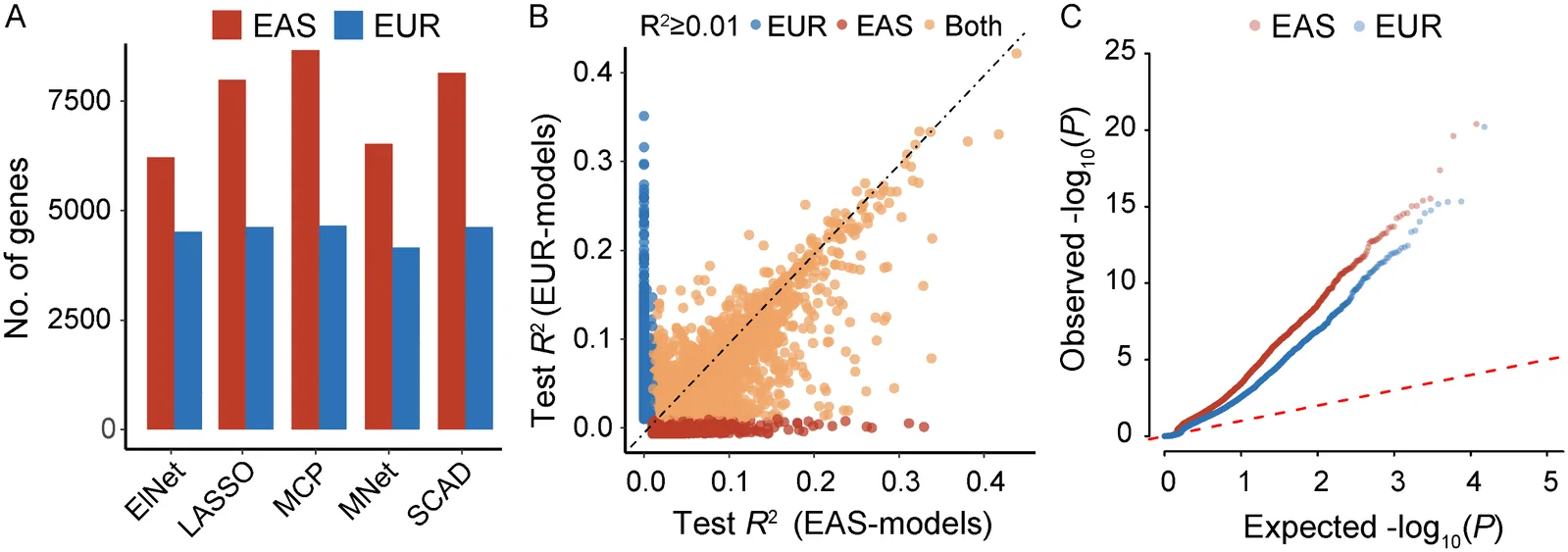
Predictive performance of gene expression models. **(A)** Comparison of the number of genes with test *R*^*2*^ > 0.01 for EAS- and EUR-specific models across five penalized regression models. **(B)** Comparison of the test *R*^*2*^ for EAS- and EUR-specific models. **(C)** QQ plot of *P* values for the correlation between observed and predicted expression. The test *R*^*2*^ and the *P* values are selected from the best model with highest *R*^*2*^ among five training methods for each gene and only genes with best model achieving test *R*^*2*^ > 0.01 are included in panels B and **C.**

To compare the performance of expression prediction models applied across ancestries, we used EUR-specific models to impute gene expression in the unmatched EAS population. For EUR models, we downloaded expression prediction models trained on the summary-level eQTL data from the eQTLGen consortium, which includes 31,684 blood samples [19]. Using the same Shiyan dataset as test data, we found that, despite the larger sample size of the expression reference panel for EUR models, qualified models developed in EUR samples did not significantly outperform qualified models developed in EAS samples across five training methods, with an improvement in the median *R*^2^ by 0 to 0.0043 only (S1 Table). Moreover, the number of qualified EAS-specific models was consistently larger than that of EUR models across all five regression methods (Fig 2A and S1 Table). By comparing the *R*^2^ of qualified models for each gene by the best method trained from EUR samples versus from EAS samples, we further observed that EAS models had higher prediction accuracy for imputing gene expression in samples matched for ancestry (Fig 2B). Additionally, a higher lambda value in correlation *P*-values for EAS models (Fig 2C) suggests that ancestry-matched models capture more significant genetic associations. These results suggested that the prediction performance of the expression model declined when applied across ancestries, even when the sample size of the expression reference panel used for training was large.

Using DNA methylation data from the DFTJ cohort, we constructed methylation prediction models for association analysis. CpG sites located within the major histocompatibility complex (MHC) region were excluded due to the complexity of linkage disequilibrium in this region. After quality control, we successfully built prediction models for a total of 249,346 CpGs. These models achieved a prediction performance of *R*² ≥ 0.01 in both the training and validation datasets and were considered for downstream association analyses using the S-PrediXcan framework [45].

### Associations of predicted gene expression with lung cancer risk

In the discovery stage, we identified 25 genes whose genetically predicted expressions were associated with lung cancer risk at FDR < 0.05 (Fig 3A). Of these, 19 genes located in 12 loci were more than 500kb away from previously reported loci (S2 Table).

**Fig 3:**
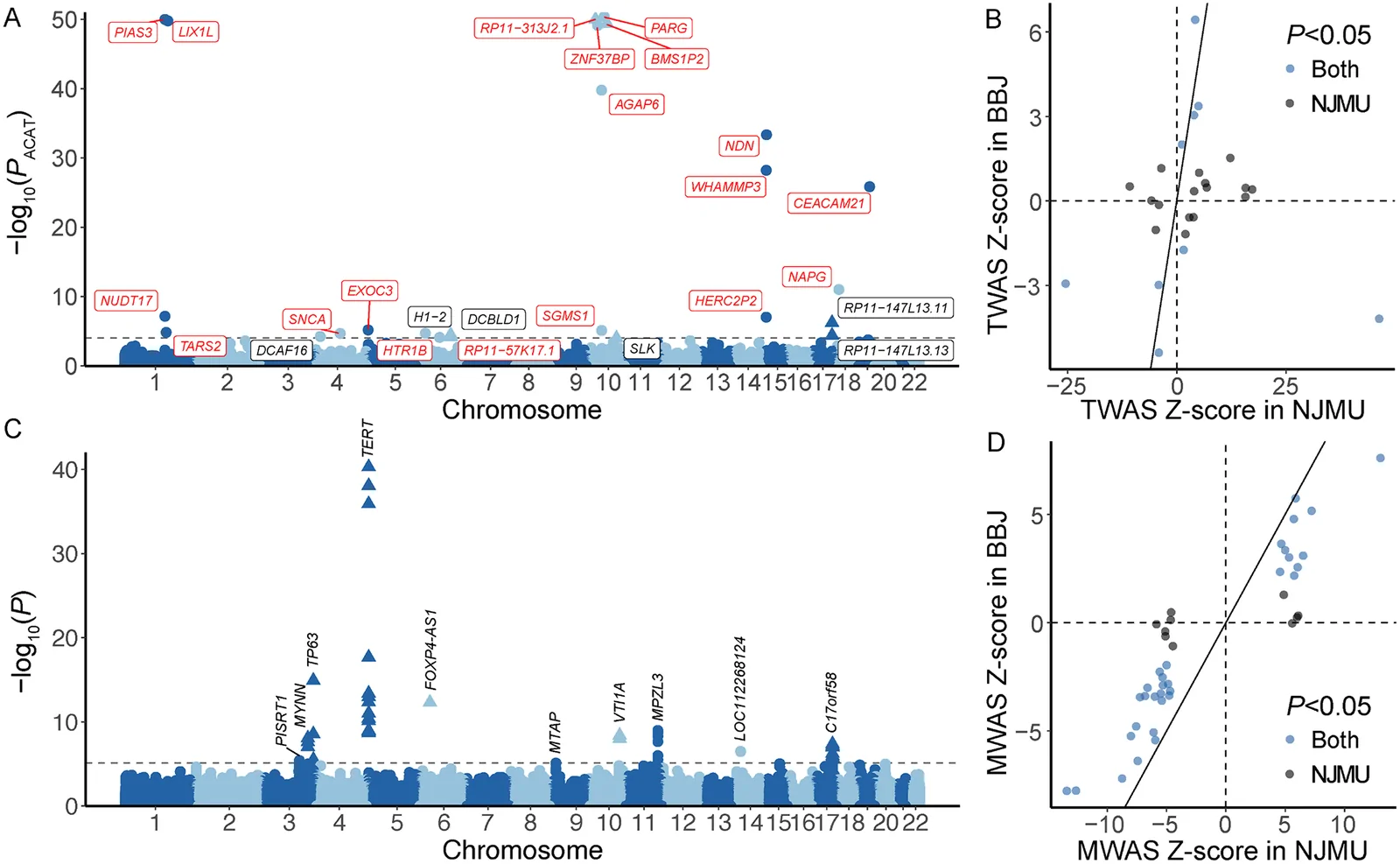
Transcriptome-wide and methylome-wide association study for lung cancer. **(A)** Manhattan plot of TWAS results in the discovery stage. Significant genes are labeled, with novel susceptibility genes highlighted in red. *P*-values represent *P*_ACAT_ across five methods with −log_10_(*P*_ACAT_) capped at 50. **(B)** Comparison of Z-scores for 25 significant genes between the discovery and replication stages. Z-scores are derived from the best prediction model for each gene. **(C)** Manhattan plot of MWAS results in the discovery stage. **(D)** Comparison of Z-scores for 42 significant CpGs between the discovery and the replication stages. The horizontal dashed line marks the FDR-corrected genome-wide significance threshold and the diamonds indicate replicated genes/CpGs in the panel A and **C.**

To further evaluate the contribution of EAS-specific prediction models, we compared TWAS results obtained using EAS- and EUR-based models in the NJMU discovery dataset. The EUR-based models tested 11,425 genes, including 5,137 genes also tested by the EAS-based models. The EUR-based TWAS identified 10 significant genes (FDR < 0.05), compared with 25 genes identified by the EAS-based TWAS (S3 Table). Among genes tested by both approaches, two were significant in both analyses, 11 were significant only using EAS-based models, and two were significant only using EUR-based models. The gene-level Z-score scatter plot showed partially overlapping but distinct association patterns between the two approaches, supporting the utility of ancestry-matched prediction models (S1 Fig).

For replication, we tested the 25 significant genes and successfully validated seven genes showing consistent effect direction and nominal significance (*P* < 0.05) (Fig 3B, Table 1). Among the top discovery hits, *RP11-313J2.1* passed the replication with a consistent positive association with lung cancer risk, while *PIAS3* failed to replicate due to an opposite effect direction (Table 1 and S2 Table). Four of the seven validated genes were located within previously reported lung cancer GWAS loci [46], including *DCBLD1* (*Z*_*meta*_ = 7.33, *P*_*meta*_ = 2.26 × 10^−13^), *SLK* (*Z*_*meta*_ = 4.99, *P*_*meta*_ = 6.20 × 10^−7^), *RP11-147L13.11* (*Z*_*meta*_ = 5.99, *P*_*meta*_ = 2.16 × 10^−9^), and *RP11-147L13.13* (*Z*_*meta*_ = -5.08, *P*_*meta*_ = 3.86 × 10^−7^). The other three were novel genes, including *RP11-313J2.1* (*Z*_*meta*_ = 2.21, *P*_*meta*_ = 2.71 × 10^−2^)*, RP11-57K17.1* (*Z*_*meta*_ = -6.57, *P*_*meta*_ = 4.91 × 10^−11^)*,* and *BMS1P2* (*Z*_*meta*_ = -21.60, *P*_*meta*_ = 1.65 × 10^−103^) (S2-S4 Figs)*.*

**Table 1:** The seven validated genes associated with lung cancer identified by transcriptome-wide association study.

Gene	Cytoband	Gene type	Best model	Discovery		Validation		Combined^c^		Coloc PP.H4
				*Z* ^a^	*P*_*ACAT*_^*b*^	*Z* ^a^	*P*_*ACAT*_^*b*^	*Z*_*meta*_	*P*_*meta*_	
*DCBLD1*	6q22.1	Protein coding	MCP	4.22	3.22 × 10 ^− 5^	6.43	1.38 × 10 ^− 10^	7.33	2.26 × 10 ^− 13^	1.000
*RP11-57K17.1* ^d^	6q22.31	Pseudogene	MCP	-4.09	6.43 × 10 ^− 5^	-5.39	9.63 × 10 ^− 8^	-6.57	4.91 × 10 ^− 11^	0.984
*RP11-313J2.1* ^d^	10q11.21	Pseudogene	MCP	1.22	0	2.00	4.51 × 10 ^− 2^	2.21	2.71 × 10 ^− 2^	0.019
*BMS1P2* ^d^	10q11.22	Pseudogene	MCP	-25.45	1.82 × 10 ^− 142^	-2.94	8.95 × 10 ^− 3^	-21.60	1.65 × 10 ^− 103^	0.068
*SLK*	10q24.33-q25.1	Protein coding	SCAD	3.95	9.79 × 10 ^− 5^	3.05	2.35 × 10 ^− 3^	4.99	6.20 × 10 ^− 7^	0.171
*RP11-147L13.11*	17q24.2	lncRNA	ElNet	4.97	5.74 × 10 ^− 7^	3.37	7.81 × 10 ^− 4^	5.99	2.16 × 10 ^− 9^	0.994
*RP11-147L13.13*	17q24.2	lncRNA	ElNet	-4.11	3.89 × 10 ^− 5^	-2.99	2.77 × 10 ^− 3^	-5.08	3.86 × 10 ^− 7^	0.946

a Z-score is derived from the best model.

b *P*_ACAT_ is the aggregated *P*-value from five gene expression prediction models.

c Results from the fixed-effect meta-analysis of the discovery stage and replication stage.

d The gene is located in novel loci.

MCP, Minimax Concave Penalty; SCAD, Smoothly Clipped Absolute Deviation; ElNet, Elastic Net;PP.H4, posterior probability that the gene and lung cancer share the same variant; lncRNA, long noncoding RNAs.

For each validated gene, we conducted colocalization analyses to estimate the posterior probability of a shared genetic variant for lung cancer and gene expression. Four of the seven genes identified in the TWAS were linked to lung cancer showing a high probability of colocalization (PP.H4 > 0.75), including *DCBLD1* at 6q22.1, *RP11-57K17.1* at 6q22.31, *RP11-147L13.11* and *RP11-147L13.13* at 17q24.2 (Table 1).

### Associations of predicted DNA methylation with lung cancer risk

In the discovery stage, 42 CpGs at 10 loci were identified as significantly associated with lung cancer risk (FDR < 0.05) (Fig 3C, S4 Table). The top association was observed for cg00624418 at *TERT,* which is a known lung cancer risk gene [51]. In the replication stage, 32 of the 42 significant CpGs were validated showing consistent effect direction and nominal significance (*P* < 0.05) (Fig 3D, Table 2). Among these, 11 CpGs exhibited positive associations with lung cancer risk, while 21 demonstrated negative associations. All 32 validated CpGs were located at known loci that were previously reported in the EAS population [46].

**Table 2:** The 32 validated CpGs associated with lung cancer identified by methylome-wide association study.

CpG	Cytoband	Closest gene	Functional consequence	Discovery		Validation		Combined^a^		Coloc PP.H4
				*Z*	*P*	*Z*	*P*	*Z*_*meta*_	*P*_*meta*_	
cg21687591	3q26.2	*MYNN*	Intronic	5.76	8.45 × 10 ^− 9^	2.17	2.98 × 10 ^− 2^	5.84	5.25 × 10 ^− 9^	1.000
cg27020690	3q26.2	*TERC*	Downstream	-5.57	2.49 × 10 ^− 8^	-2.27	2.34 × 10 ^− 2^	-5.76	8.67 × 10 ^− 9^	1.000
cg06634950	3q26.2	*ACTRT3*	Exonic	-5.34	9.22 × 10 ^− 8^	-2.52	1.17 × 10 ^− 2^	-5.74	9.76 × 10 ^− 9^	1.000
cg23118535	3q28	*TP63*	Intronic	-8.01	1.18 × 10 ^− 15^	-5.24	1.64 × 10 ^− 7^	-9.52	1.80 × 10 ^− 21^	0.998
cg14805663	3q28	*TP63*	Intronic	-5.94	2.81 × 10 ^− 9^	-5.42	5.83 × 10 ^− 8^	-8.03	9.57 × 10^-16^	0.002
cg21214753	3q28	*TP63*	Intronic	4.67	3.00 × 10 ^− 6^	3.64	2.72 × 10 ^− 4^	5.92	3.21 × 10 ^− 9^	0.000
cg20909875	3q28	*TP63*	Intronic	4.56	5.01 × 10 ^− 6^	2.34	1.92 × 10 ^− 2^	5.02	5.20 × 10 ^− 7^	0.000
cg00624418	5p15.33	*TERT*	Intronic	-13.41	5.12 × 10 ^− 41^	-7.77	8.12 × 10 ^− 15^	-15.31	6.99 × 10 ^− 53^	0.000
cg23211257	5p15.33	*TERT*	Intronic	13.03	8.60 × 10 ^− 39^	7.60	3.04 × 10^-14^	14.90	3.29 × 10 ^− 50^	0.873
cg13924604	5p15.33	*TERT*	Intronic	-12.64	1.23 × 10 ^− 36^	-7.75	9.11 × 10 ^− 15^	-14.70	6.51 × 10 ^− 49^	0.000
cg17166338	5p15.33	*TERT*	Upstream	-8.75	2.09 × 10 ^− 18^	-7.19	6.29 × 10 ^− 13^	-11.33	9.37 × 10 ^− 30^	0.000
cg08894401	5p15.33	*CLPTM1L*	Intronic	-7.56	4.02 × 10 ^− 14^	-4.79	1.68 × 10 ^− 6^	-8.89	6.29 × 10^-19^	0.000
cg11666982	5p15.33	*TERT*	Exonic	-7.43	1.06 × 10 ^− 13^	-6.38	1.73 × 10 ^− 10^	-9.80	1.19 × 10 ^− 22^	0.000
cg25045012	5p15.33	*TERT*	Intronic	-7.24	4.45 × 10 ^− 13^	-3.44	5.84 × 10 ^− 4^	-7.79	6.82 × 10 ^− 15^	0.000
cg09508356	5p15.33	*CLPTM1L*	Upstream	-6.81	9.52 × 10 ^− 12^	-3.39	7.04 × 10 ^− 4^	-7.42	1.13 × 10 ^− 13^	0.000
cg26947723	5p15.33	*CLPTM1L*	Intronic	-6.61	3.94 × 10 ^− 11^	-3.00	2.68 × 10 ^− 3^	-7.02	2.22 × 10 ^− 12^	0.000
cg27491040	5p15.33	*MIR4457; CLPTM1L*	Intergenic	6.51	7.31 × 10 ^− 11^	3.09	2.00 × 10 ^− 3^	7.00	2.49 × 10 ^− 12^	0.000
cg27311041	5p15.33	*TERT; MIR4457*	Intergenic	-6.10	1.05 × 10 ^− 9^	-5.07	4.07 × 10 ^− 7^	-7.93	2.20 × 10 ^− 15^	0.000
cg26522247	5p15.33	*LINC01511*	ncRNA intronic	6.06	1.39 × 10 ^− 9^	2.56	1.05 × 10 ^− 2^	6.31	2.73 × 10 ^− 10^	0.000
cg25070340	5p15.33	*CLPTM1L*	Intronic	-5.99	2.07 × 10 ^− 9^	-3.41	6.41 × 10 ^− 4^	-6.80	1.03 × 10 ^− 11^	0.000
cg04073127	6p21.1	*FOXP4-AS1*	ncRNA intronic	7.23	4.77 × 10 ^− 13^	5.16	2.45 × 10 ^− 7^	8.87	7.51 × 10 ^− 19^	1.000
cg16024028	10q25.2	*VTI1A*	Intronic	5.88	4.07 × 10 ^− 9^	5.74	9.54 × 10 ^− 9^	8.18	2.75 × 10 ^− 16^	1.000
cg27253415	10q25.2	*VTI1A*	Intronic	5.73	9.97 × 10 ^− 9^	4.78	1.71 × 10 ^− 6^	7.47	8.32 × 10 ^− 14^	1.000
cg17657885	17q24.2	*C17orf58; KPNA2*	Intergenic	-5.48	4.16 × 10 ^− 8^	-3.27	1.07 × 10 ^− 3^	-6.32	2.63 × 10 ^− 10^	0.998
cg25512481	17q24.2	*C17orf58*	Intronic	-5.41	6.47 × 10 ^− 8^	-3.60	3.17 × 10 ^− 4^	-6.47	1.01 × 10 ^− 10^	0.999
cg06855216	17q24.2	*C17orf58; KPNA2*	Intergenic	5.33	9.62 × 10 ^− 8^	3.01	2.62 × 10 ^− 3^	6.04	1.57 × 10 ^− 9^	0.990
cg22429852	17q24.2	*C17orf58; KPNA2*	Intergenic	-5.31	1.10 × 10 ^− 7^	-2.89	3.82 × 10 ^− 3^	-5.94	2.78 × 10 ^− 9^	0.993
cg00081098	17q24.2	*NOL11; BPTF*	Intergenic	-5.00	5.69 × 10 ^− 7^	-1.96	4.99 × 10 ^− 2^	-5.12	3.10 × 10 ^− 7^	1.000
cg04849995	17q24.2	*C17orf58; KPNA2*	Intergenic	5.00	5.81 × 10 ^− 7^	3.35	8.23 × 10 ^− 4^	5.99	2.13 × 10 ^− 9^	0.989
cg19720302	17q24.2	*C17orf58*	Intronic	-4.87	1.11 × 10^− 6^	-2.83	4.71 × 10 ^− 3^	-5.56	2.66 × 10 ^− 8^	0.999
cg12739664	17q24.2	*C17orf58*	Intronic	-4.78	1.73 × 10^− 6^	-3.36	7.79 × 10 ^− 4^	-5.83	5.54 × 10 ^− 9^	1.000
cg12091567	17q24.2	*LINC00674*	ncRNA exonic	-4.70	2.56 × 10^− 6^	-3.15	1.61 × 10 ^− 3^	-5.64	1.71 × 10 ^− 8^	0.993

^a^Results from the fixed-effect meta-analysis of the discovery stage and replication stage. PP.H4, posterior probability that the CpG site and lung cancer risk share the same variant; ncRNA, noncoding RNA.

To further investigate the MWAS findings, we performed colocalization analyses at these 32 validated CpGs using the DFTJ mQTL data (N = 4,411) and the results of the lung cancer GWAS meta-analysis. The results indicated that 17 CpGs shared common causal variants with lung cancer risk (PP.H4 > 0.75), highlighting their potential roles in epigenetic regulation (Table 2).

### Three-step summary data-based Mendelian randomization

For the gene-lung cancer SMR analysis, we tested the associations between six TWAS-identified genes and lung cancer risk after excluding one gene without significant eQTL (*P*_eQTL_ < 1 × 10^−5^). Four MR associations were detected (Bonferroni-corrected *P*_SMR_ < 0.05), including two genes (*SLK* and *RP11-147L13.11*) that passed the HEIDI test with *P*_HEIDI_ > 0.01 (S5 Table).

For the CpG-lung cancer SMR analysis, we prioritized the 32 MWAS-identified CpGs using the DFTJ mQTL data. A total of 15 CpGs were found to be significant in the SMR analysis with Bonferroni-corrected *P*_SMR_ < 0.05 and *P*_HEIDI_ > 0.01 (S6 Table).

Furthermore, we explored the associations between the 32 CpGs identified by MWAS and the six genes identified by TWAS using a CpG-gene SMR analysis. We detected eight CpGs associated with two genes (Bonferroni-corrected *P*_SMR_ < 0.05, *P*_HEIDI_ > 0.01, S7 Table). Notably, the association of cg19720302 and cg04849995 and their assumed target gene *RP11-147L13.11* were significant in all MR analyses, providing strong evidence of plausible regulatory mechanisms (Fig 4). Additionally, the gene closest to the top eQTL rs78192857 is not *RP11-147L13.11*, indicating potential long-range regulatory effects (Fig 4). These findings suggested that genetic variants may influence lung cancer risk by modulating gene expression through alterations in the methylation levels.

**Fig 4:**
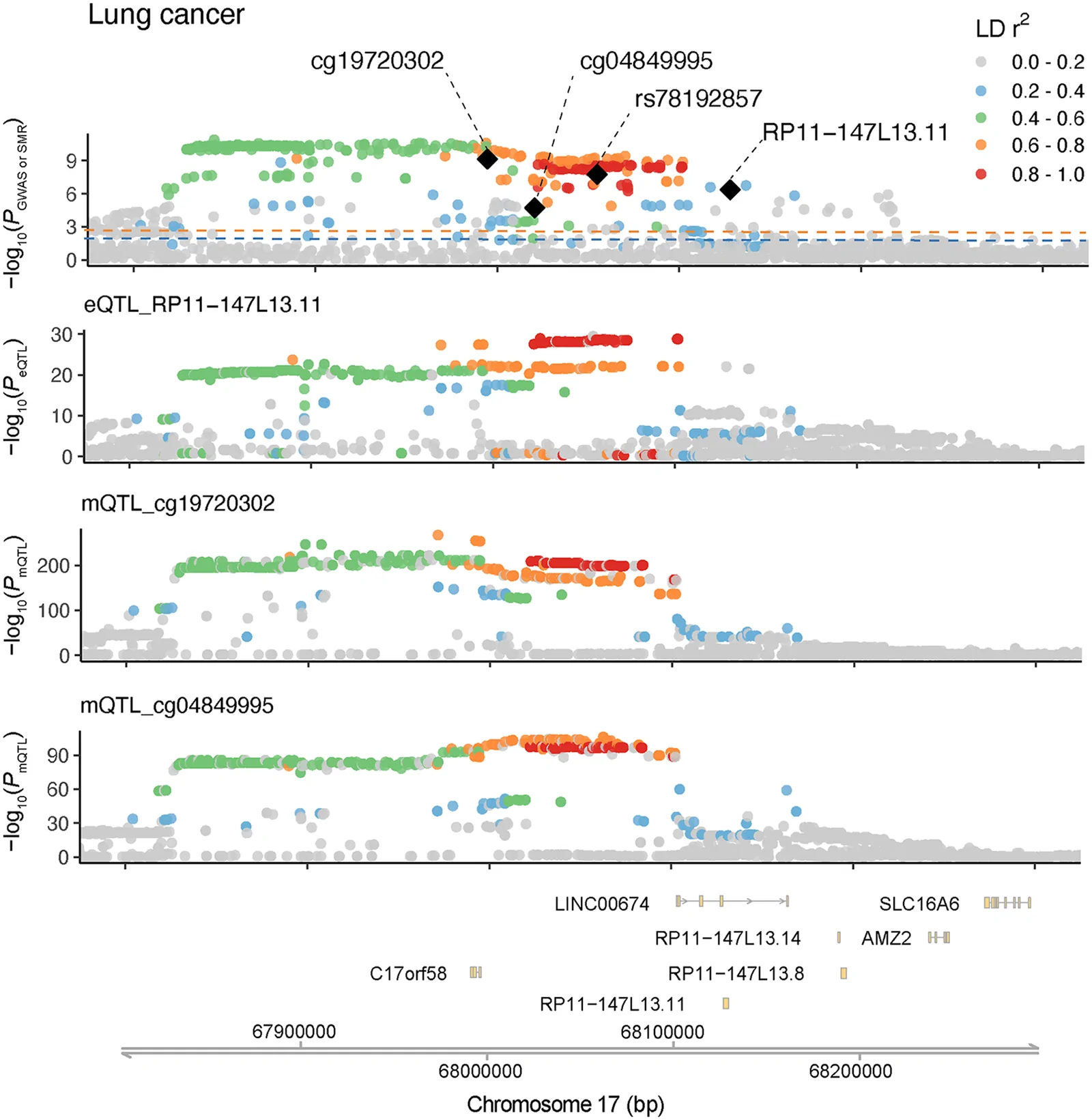
Prioritizing genes and regulatory elements at the *RP11-147L13.11* locus for lung cancer. Results of the three-step SMR analysis that integrates data from GWAS, eQTL, and mQTL studies are shown. The top panel shows −log_10_*P* of SNPs from the GWAS meta-analysis for lung cancer. Black diamonds highlight the SMR results for cg19720302, cg04849995, and *RP11-147L13.11*, as well as the GWAS result for rs78192857. The blue and orange dashed lines indicate the significance thresholds for the gene-lung cancer and CpG-lung cancer SMR analyses, respectively. The second panel shows −log_10_*P*_eQTL_ of SNPs for *RP11-147L13.11* in the EAS eQTL meta-analysis. The third and fourth panels show −log_10_*P*_mQTL_ of SNPs for cg19720302 and cg04849995 in the DFTJ mQTL study. rs78192857, the top cis-eQTL for RP11-147L13.11, was used as the index SNP and all other SNPs are colored according to their linkage disequilibrium (LD; r²) with rs78192857. Gene annotations and genomic positions on chromosome 17 are shown at the bottom.

## Discussion

In this study, we conducted a two-stage TWAS and MWAS of lung cancer based on EAS-specific gene expression and methylation prediction models. Seven genes and 32 CpGs were identified to be associated with lung cancer risk, with four genes and all CpGs mapped to known GWAS loci. The remaining three genes (*RP11-313J2.1, RP11-57K17.1, BMS1P2*) represent potential novel candidate loci with no prior reported association with lung cancer. Notably, the three-step SMR analysis highlighted a novel regulatory pathway of *RP11-147L13.11* that was influenced by DNAm levels of cg04849995 and cg19720302. These findings corroborate previous studies and provide new insights into the molecular mechanisms of lung cancer.

Compared with previous TWAS and MWAS of lung cancer [13,52,53], this study developed gene expression and DNA methylation prediction models using the largest available EAS eQTL and mQTL datasets, enabling a more comprehensive identification of susceptibility loci. These prediction models enabled us to identify lung cancer-associated gene expression and DNA methylation specific to EAS ancestry. Among the seven genes identified by TWAS, *DCBLD1* (6q22.1) and *SLK* (10q24.33-q25.1) were previously found to be associated with lung cancer risk by GWAS [54,55]. *DCBLD1*, which is involved in focal adhesions and cell migration, plays a role in lung cancer progression [56]. *SLK*, the Ste20-like kinase, participates in cytoskeletal dynamics and has demonstrated both tumor-suppressive and promotive properties in an interaction-dependent manner [57]. Although *SLK* was identified in both TWAS and previous GWAS studies, the relatively low colocalization support and borderline HEIDI test suggest that the observed association may not be mediated through blood gene expression. *RP11-147L13.11* and *RP11-147L13.13* are lncRNA genes located in previously established lung cancer GWAS loci[5], and have not been previously studied in the context of lung cancer. In addition, these four genes all had colocalization evidence for lung cancer risk. The remaining three genes, located in previously unidentified loci, are all classified as pseudogenes. Although pseudogenes were once considered non-functional, emerging evidence suggests they may play regulatory roles in tumorigenesis through competitive endogenous RNAs (ceRNAs) mechanisms or other pathways [58,59]. *RP11-57K17.1* (6q22.31), a pseudogene of *DDX24*, showed higher expression linked to reduced lung cancer risk, potentially through the downregulation of its parental gene. This hypothesis was consistent with the reported pro-tumorigenic role of *DDX24* in non-small cell lung cancer [60]. *RP11-313J2.1* (10q11.21), a pseudogene of a zinc finger protein, may indirectly regulate zinc finger protein expression, which is frequently involved in oncogenesis, including lung cancer [61]. *BMS1P2* (*BMS1* pseudogene 2) at 10q11.22 is involved in the ribosome biogenesis pathway [62], the dysregulation of which has been implicated in cancer progression [63]. However, *RP11-313J2.1* and *BMS1P2* showed limited colocalization support and therefore should be interpreted cautiously pending further validation.

Several EWASs [21, 23] and one MWAS have been conducted for lung cancer [64] in both EUR and EAS populations. However, our EAS-specific MWAS only replicated two CpGs (cg04849995, cg19720302) from previous findings [64]. The inability to reproduce most CpGs in EWASs may be attributed to the variability of DNAm influenced by the environment. Additionally, the limited overlap with prior MWAS [52] may result from potential biases introduced by applying cross-ancestry methylation prediction models in the earlier study. We overcame these limitations by using EAS-specific methylation prediction models, which align with the population structure of the lung cancer GWAS data. Moreover, we constructed methylation prediction models using the Illumina EPIC array, which provides broader CpG coverage compared to models constructed using the Illumina 450k array. Importantly, 15 of the 32 CpGs identified in our MWAS were unique to the EPIC array, suggesting that we could have identified additional regulatory sites not captured in previous studies. Besides, all 32 CpGs were located near previously GWAS reported lung cancer susceptibility loci, suggesting that MWAS may provide additional insights into how genetic susceptibility loci influence lung cancer risk through DNA methylation regulation. Notably, cg19720302 and cg04849995, located at 17q24.2, were found to influence *RP11-147L13.11* expression in a direction consistent with TWAS and MWAS signals, suggesting a potential regulatory pathway warranting further validation.

Several limitations of this study should be acknowledged. First, while the eQTL dataset for constructing gene expression models was one of the largest available for the EAS population, its sample size remains relatively small compared to the EUR eQTL datasets. This may limit the statistical power to detect smaller genetic effects. Recently developed multi-ancestry transcriptome prediction approaches, such as mashr [64] and sushie [65], may further improve cross-population prediction performance by leveraging shared genetic architecture across ancestries. Future studies integrating these methods with larger EAS eQTL resources may help further improve transcriptome prediction accuracy and statistical power. In addition, due to the difficulty in obtaining lung tissue samples from East Asian populations, the prediction models in this study were primarily constructed using whole blood and leukocyte datasets. Therefore, our analyses may not fully capture lung tissue-specific gene expression and DNA methylation regulatory patterns related to lung cancer susceptibility. Second, the replication stage used a nominal significance threshold (*P* < 0.05) rather than a more stringent multiple-testing corrected threshold, which may have increased the possibility of false-positive findings. Third, the associations between the two genes and lung cancer risk, and between the 15 CpGs and lung cancer risk, as identified by MR analyses, are potentially pleiotropic and should be interpreted with caution, as they do not constitute conclusive causal evidence. Lastly, the JCTF eQTL dataset used in our expression reference panel was derived from a case-control study of COVID-19 [29], which may introduce differences in expression profiles relative to those of healthy individuals. However, according to the previous study, the JCTF eQTL dataset is still considered a reliable reference dataset with minimal overall impact from the disease status of the samples [29]. Future studies employing larger sample sizes of healthy EAS individuals are necessary to improve model accuracy and validate our findings.

In conclusion, this study identified seven genes through TWAS and 32 CpGs through MWAS that are associated with lung cancer risk in the EAS population. Integration of multi-omics data revealed regulatory pathways through which cg19720302 and cg04849995 modulated *RP11-147L13.11* in the tumorigenesis of lung cancer. Our findings enhance the understanding of lung cancer genetics and emphasize the utility of multi-omics integration in identifying novel cancer biomarkers and potential regulatory mechanisms.

## Supporting information

S1 Table Comparison of test R2 of EAS-specific models and EUR-specific models in the Shiyan dataset. (XLSX)

S2 Table Lung cancer-associated genes identified by transcriptome-wide association study. (XLSX)

S3 Table Comparison of significant TWAS genes identified using EAS- and EUR-based prediction models in the NJMU discovery dataset. (XLSX)

S4 Table Lung cancer-associated CpG identified by methylome-wide association study. (XLSX)

S5 Table Prioritized genes for lung cancer identified from the SMR analysis. (XLSX)

S6 Table Prioritized CpGs for lung cancer identified from the SMR analysis. (XLSX)

S7 Table Mapping the lung cancer-associated CpGs to the lung cancer-associated genes by the SMR analysis. (XLSX)

S1 Fig Comparison of gene-level TWAS *Z*-scores between EAS- and EUR-based prediction models in the NJMU discovery dataset. Each point represents a gene tested by both EAS- and EUR-based prediction models. The x-axis shows TWAS *Z*-scores obtained using EUR-based models, and the y-axis shows TWAS *Z*-scores obtained using EAS-based models. Genes are colored according to their significance status at FDR < 0.05: grey, not significant in either analysis; blue, significant only using EAS-based models; yellow, significant only using EUR-based models; pink, significant in both analyses.
(TIF)

S2 Fig Regional association plots for the *RP11-57K17.1* locus. The upper panels show lung cancer GWAS association signals, the middle panels show eQTL association signals for the *RP11-57K17.1* in the EAS eQTL meta-analysis.
(TIF)

S3 Fig Regional association plots for the *RP11-313J2.1* locus. The upper panels show lung cancer GWAS association signals, the middle panels show eQTL association signals for the *RP11-313J2.1* in the EAS eQTL meta-analysis.
(TIF)

S4 Fig Regional association plots for the *BMS1P2* locus. The upper panels show lung cancer GWAS association signals, the middle panels show eQTL association signals for the *BMS1P2* in the EAS eQTL meta-analysis.
(TIF)
